# Oviposition and Embryotoxicity of *Indigofera suffruticosa* on Early Development of *Aedes aegypti* (Diptera: Culicidae)

**DOI:** 10.1155/2012/741638

**Published:** 2011-07-28

**Authors:** Jeymesson Raphael Cardoso Vieira, Roberta Maria Pereira Leite, Izabela Rangel Lima, Daniela do Amaral Ferraz Navarro, Everson Miguel Bianco, Sônia Pereira Leite

**Affiliations:** ^1^Departamento de Histologia e Embriologia, Centro de Ciências Biológicas da Universidade Federal de Pernambuco, Cidade Universitária, 50670-420 Recife, PE, Brazil; ^2^Departamento de Química Fundamental, Centro de Ciências Exatas e da Natureza da Universidade Federal de Pernambuco, Cidade Universitária, 50740-560 Recife, PE, Brazil; ^3^Laboratório de Cultura de Células II—Departamento de Histologia e Embriologia, Universidade Federal de Pernambuco (UFPE), Cidade Universitária, 50670-420 Recife, PE, Brazil

## Abstract

Aqueous extract of *Indigofera suffruticosa* leaves obtained by infusion was used to evaluate the oviposition, its effect on development of eggs and larvae, and morphological changes in larvae of *Aedes aegypti*. The bioassays were carried out with aqueous extract in different concentrations on eggs, larvae, and female mosquitoes, and the morphological changes were observed in midgut of larvae. The extract showed repellent activity on *A. aegypti* mosquitoes, reducing significantly the egg laying by females with control substrate (343 (185–406)) compared with the treated substrate (88 (13–210)). No eclosion of *A. aegypti* eggs at different concentrations studied was observed. The controleclodedin 35%. At concentration of 250 **μ**g/mL, 93.3% of larvae remained in the second instar of development and at concentrations of 500, 750, and 1000 **μ**g/mL the inhibitory effect was lower with percentages of 20%, 53.3%, and 46.6%, respectively. Morphological changes like disruption on the peritrophic envelope (PE), discontinued underlying epithelium, increased gut lumen, and segments with hypertrophic aspects were observed in anterior region of medium midgut of larvae of *A. aegypti*. The results showed repellent activity, specific embryotoxicity, and general growth retardation in *A. aegypti* by medium containing aqueous extract of *I. suffruticosa* leaves.

## 1. Introduction

The mosquito *Aedes aegypti* Linnaeus is a vector and promotes the spreading of four serotypes of dengue virus. However, a decrease in the effective vector control has been described due to larval tolerance to chemical insecticides [1]. The incidence of classical and hemorrhagic dengue fever in 2007 registered by the Brazilian Federal Organ was 559 954 cases, with 158 deaths in the country [2]. Despite significant advances in the techniques used for its control during recent decades, the mosquito *A. aegypti* continues to pose serious public health problems [3]. A dengue vaccine is still under development, and vector control is the only practical measure towards the reduction of dengue disease [1]. 

It has been demonstrated that insect gut is the target of many insecticidal compounds. Transmission electron microscopy of *A. aegypti* larvae treated with an aqueous extract of *Derris urucu* showed histological alterations in the midgut, and larval mortality was associated with peritrophic matrix damage [4]. The peritrophic matrix of insects is constituted by proteins, glycoproteins, proteoglycans, and chitin, and its integrity is important for digestive processes as well as for protection against invasion by microorganisms and parasites [5]. Plants have been evaluated as sources of natural insecticides against *A. aegypti*, and larvicidal bioassays have been conducted using third (L3) and fourth (L4) instars or comparing the effect of plant extracts on larval development of L1–L4 [6]. Various studies have addressed the possibility of using the embryo culture technique as an assay for embryotoxic potential of xenobiotic compounds [7].

*Indigofera suffruticosa* Mill (Fabaceae) is a plant found in tropical and subtropical areas and well adapted to growth in semiarid regions and soil of low fertility [8]. This plant occurs in Brazil Northeast countryside and has intensive popular use in the treatment of bacterial and fungi infections, inflammations, and other diseases such as epilepsy in human and animal models [9, 10]. In Brazil, the plants have been used as an infusion or decoct (flavor extract by boiling 1 L of hot water/5 g of leaves) [9].

A chemical investigation of this species (*I. suffruticosa*) in Natural Products Alert (NAPRALERT) [11] and Chemical Abstracts databases has revealed the presence of alkaloids, flavanoids, steroids, proteins, carbohydrates, and indigo.

Recently, antitumoral and antimicrobial activities and mice embryotoxic effects have been tested with extract of leaves of *I. suffruticosa* [10, 12–14].

In the present study, we have investigated the process of oviposition, early development on eggs and larvae of *A. aegypti*, and morphological changes in larvae treated with aqueous extract from leaves of *I. suffruticosa.*

## 2. Materials and Methods

### 2.1. Plant Material

The leaves of *I. suffruticosa* were collected in October 2005 in Igarassu, State of Pernambuco, Brazil, and authenticated by the Biologist Marlene Barbosa from the Botanic Department, Universidade Federal de Pernambuco (UFPE). A voucher specimen number 32859 has been deposited at the Herbarium of the above-cited department.

### 2.2. Mosquitoes

Eggs and larvae of *A. aegypti* were originally obtained from Centro de Vigilância Ambiental da Prefeitura Municipal do Recife, Pernambuco, Brazil, and female mosquitoes from the ecology laboratory of Chemistry Department of Universidade Federal de Pernambuco/UFPE. Adult mosquitoes (F0 generation) were fed with 10% glucose and with chicken blood and were reared in a room maintained at 27°C in humidified cages. Eggs of these mosquitoes were counted using a stereoscopic microscope. The larvae generated were fed with commercial cat food. Eggs and the 1st instars larvae were used in the experiments.

### 2.3. Preparation of the Extracts

Leaves (75 g) were weighed and chopped. The plant material was successively extracted in infusion with solvents of increasing polarity (hexane, ethyl acetate, and methanol). The solvents were removed by rotary evaporation. The percentage yields were hexane (0.67%), ethyl acetate (0.39%) methanol (3.9%), and (w/w) in terms of newly collected plant material. After the extraction processes with the aforementioned solvents, the same plant material was extracted with distilled water, resulting in the aqueous extract. To the egg-laying evaluation, 25 mL of aqueous extract was used with female mosquitoes. The other part of extract was lyophilized, and the dried powder plant material (4.2%) was stored at 20°C. This dry residue aqueous extract was homogenized using 100 *μ*L of distilled water in microcentrifuge tubes, then diluting in water to the appropriate concentration 250, 500, 750, and 1000 *μ*g/mL to evaluate the embryotoxicity on eggs and larvae.

### 2.4. Oviposition Bioassay

During 4 consecutive days, 90 female mosquitoes of *A. aegypti* were stored in polypropylene cages (30 × 30 × 30 cm) (Bugdorm-I, Mega View Science Education Services, Taiwan) with sacarose solution 10% at 25°C. Females were exposed to 18 substrates (paper filter) with distilled water (9 substrates) and 25 mL at 30% of aqueous extract of *I. suffruticosa* (9 substrates). The quantification of the eggs was assessed by observation under a stereomicroscope (1.2x). The oviposition bioassay was assayed as recommended by the World Health Organization [15].

### 2.5. Embryotoxicity Bioassay

*Aedes aegypti* L., whose common name is dengue mosquito, belongs to the Arthropoda Phylum, Hexapoda Class, Diptera Order, and Culicidae Family. The effect of aqueous extract of *I. suffruticosa *leaves on egg outbreak and larval development of *A. aegypti* was assayed as recommended by the World Health Organization [15]. Eggs and larvae of *A. aegypti* were exposed to the extract in concentrations of 250, 500, 750, and 1000 *μ*g/mL. Preliminary bioassay was performed using 40 eggs that were hatched in mineral water (200 mL) at 26°C–28°C. The test using larvae (*n* = 15, 1st instar) were carried out in duplicate for each concentration. Larvae were placed into 200 mL disposable plastic cups containing 25 mL of the test solution and incubated at 27°C. The developmental stages of larvae was determined at the start of the experiment (0 h) and 24, 48, and 72 h thereafter, and developmental stages were assessed by observation under a stereomicroscope (1.2x).

#### 2.5.1. Morphologic Study of A. aegypti Larvae

Mosquito (*A. aegypti*) larvae from control and treated groups were fixed with formaldehyde (2.5%) for morphologic evaluation and were photographed using a digital video camera (Leica) connected to an inverted microscope (magnification of 200x.).

### 2.6. Statistical Analysis

We used Mann-Whitney (*P* < 0.001) using the SigmaStat (3.5 version) between the control and tested groups. The oviposition results were expressed in media (min-max).

## 3. Results

### 3.1. Oviposition Bioassay

**Figure 1:**
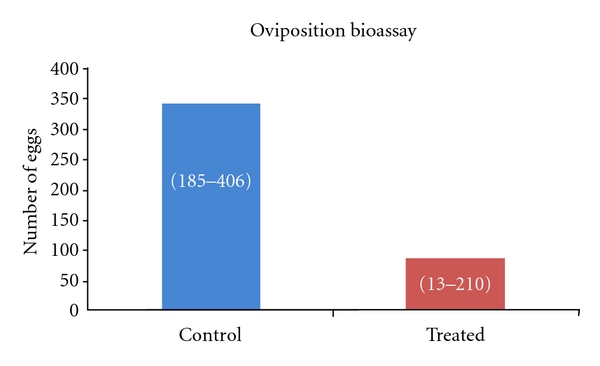
Repellent effect of aqueous extract of leaves of *I. suffruticosa* on female mosquitoes of *A.aegypti *during 4 days of observation of egg postures. Control substrate on distilled water (343 (185–406)) compared with the treated substrate on aqueous extract (88 (13–210)). The results of the oviposition test are expressed as the median (min–max). *n *= 3.634 eggs. *P < 0.001.

In the oviposition test, the mosquitoes of *A. aegypti* (90 females) the eggs were quantified (3.634 eggs) after 4 days using 30% of aqueous extract of *I. suffruticosa*. The substrate containing aqueous extract reduced significantly the posture of eggs (88 (13–210)), compared with the control treated with distilled water (343 (185–406)) (Figure 1).

### 3.2. Embryotoxicity Bioassay

No eclosion of *A. aegypti* eggs in the different concentrations studied was observed. The same number of eggs (*n* = 40) was used as a control that ecloded in 35% (Table 1).

The embryonic development of larvae of first instar (L1) of *Aedes aegypti* was observed from 0 to 72 h using concentrations from 250 to 1000 *μ*g/mL of aqueous extract of *I. suffruticosa* leaves.  Table 2 compares the effect of extract of *I. suffruticosa* at different concentrations.

Approximately 93.3% of live larvae treated with 250 *μ*g/mL of extract stopped at second instar (L2) similarly to other concentrations (550, 750, and 1000 *μ*g/mL), in which the inhibitory effect was lower with percentages of 20%, 53.3%, and 46.6%, respectively.

#### 3.2.1. Morphologic Study of A. aegypti Larvae

**Figure 2:**
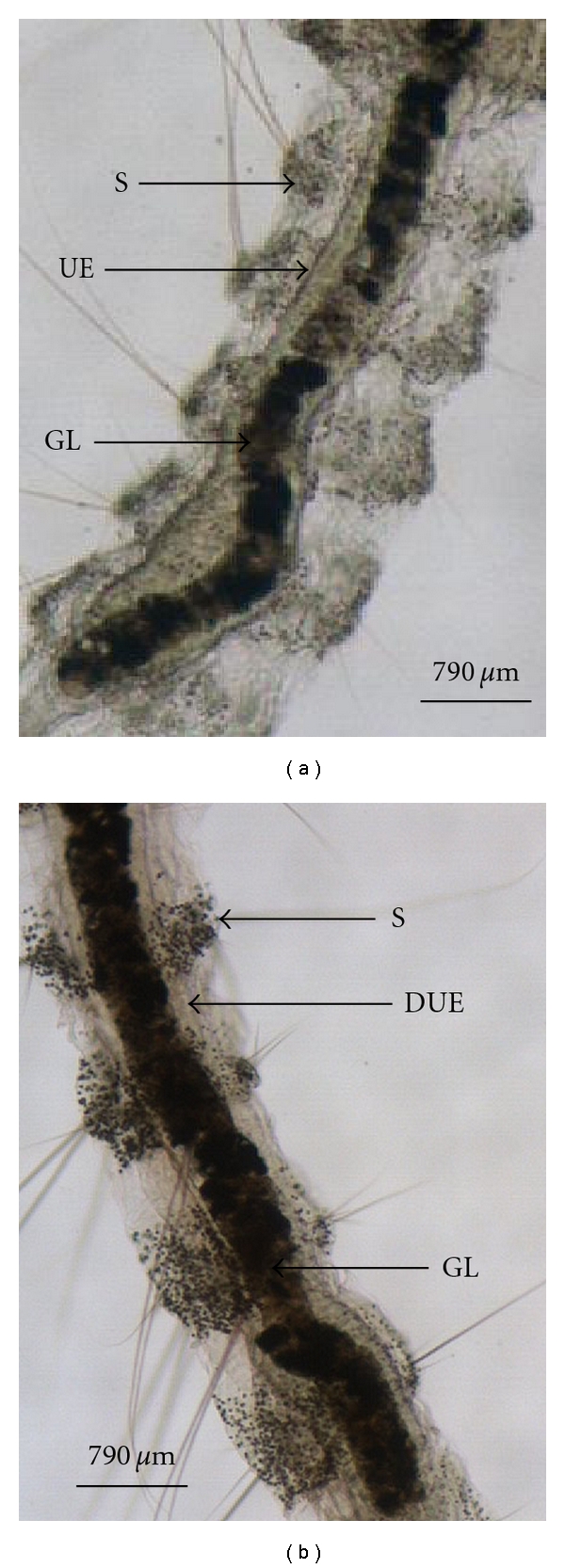
*Aedes aegypti* photomicrography (200x) of anterior midgut from live larvae after 72 h of incubation. (a) Control live L2 on distilled water. (b) Treated live L2 on aqueous extract of *I. suffruticosa*. GL, gut lumen; UE, underlying epithelium; DUE, discontinued underlying epithelium; S, segments.

Control Live L2 on distilled water (Figure 2(a)) and treated live L2 on aqueous extract of *I. suffruticosa* (Figure 2(b)) after 72 h of incubation were evaluated using inverted optical microscope. Morphological observation of anterior region of medium midgut of larvae of* Aedes aegypti* in early development treated with aqueous extract of *I. suffruticosa* showed disruption on the peritrophic envelope (PE) structure consequently resulting in a discontinued underlying epithelium, increased gut lumen, and segments with hypertrophic aspects in comparison with control larvae. The developmental delay is directly dependent of morphological changes that occur when the larvae are growing in contact with different substances of the extract.

## 4. Discussion

The purpose of this study was to determine the repellent and toxic effects of *Indigofera suffruticosa* on oviposition and embryonic development of *Aedes aegypti*. 

The results showed significant repellent effect on egg posture and specific embryotoxicity and general growth retardation on *A. aegypti* by medium containing aqueous extract of *I. suffruticosa* leaves. 

Studies reporting repellent effect with *Indigofera* species were not found in literature, but many plants from the family Lamiaceae are toxic for insects including *Ocimum basilicum, O. gratissimum, O. americanum, Cymbopogom nardus, Alpinia galanga, Syzyaium aromaticum *e *Thymus vulgaris, Mentha, Eucalyptus maculata citriodon, *and* Tagetus e Lantana camara*, and they have been studied as natural alternative repellents [16].

*A. aegypti* eggs did not outbreak and larvae in early development showed an increase of abnormalities, mainly in the peritrophic envelops at different concentrations. At 250 *μ*g/mL concentration the extract could affect one of the phases of the life cycle of *A. aegypti*. Higher incidences of specific embryotoxicity were found at concentrations that also caused general growth retardation [15]. The in vitro counterpart of teratogenicity was defined as specific embryotoxicity that could be distinguished from general retardation of growth and development of the embryo. By using this definition, general toxic effects are not considered to indicate specific embryotoxicity, since general toxicity will be induced by virtually any compound if added at sufficiently high concentrations [15]. Four compounds tested that were not teratogenic *in vivo*: amaranth [17] and isoniazid [18] had only growth retarding and/or lethal effects at high concentrations *in vitro*, whereas penicillin [19] and saccharin [20] did not show any effect at the highest concentration tested in culture. However, the most important confounding factor in the use of whole embryo culture as a screening test is likely to be the experimenter's judgment regarding the scoring of specific embryotoxicity, especially the distinction between specific toxicity, on the one hand, and general toxicity and growth retardation on the other hand. The interpretation of malformed and retarded embryos is complicated further when effects occur at low incidences, as described in the present study for extract of *I. suffruticosa*. Aqueous extract of *I. suffruticosa* leaves was studied for adverse effects in preimplantation mouse embryos. Two-cell mouse embryos were cultured for 94 h in human tubal fluid medium (HTF), and the extract at a concentration of 5 mg/mL showed a development from morula to blastocyst stages similar to the controls, and at a higher concentration (10 mg/mL), all embryos persisted at the two-cell stage [12].

In vertebrates, mucus is the primary secreted layer, lining and protecting the intestinal epithelium, while assisting the digestion process [21]. However, insects do not possess a typical mucus layer in the digestive tract, and instead, their midgut is lined by a unique protective structure, the peritrophic envelop (PE) [22]. The PE is a mucinous structure, which is uniquely different from vertebrate mucus by its incorporation of chitin, resulting in proteinaceous structure reinforced by chitin fibrils [23]. Despite these important functions, the biochemical properties and molecular biology of PE formation is still poorly understood [23].

**Figure 3:**
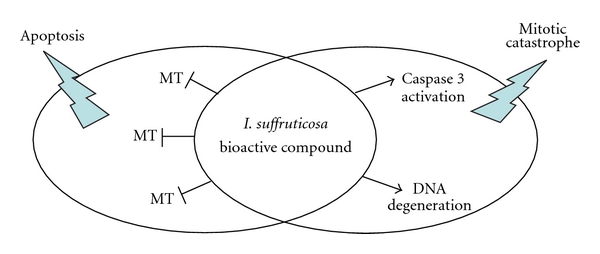
Main targets of *I. suffruticosa* bioactive compound that lead to two types of cell death. Flashes indicate the main biological cell death. MT: molecular target.

This experimental study demonstrated that extract could act promoting morphological changes on PE in larvae of *A. aegypti*. Furthermore, the inhibition of PE formation severely affected the early development of larvae. In controlling second instar larvae of *A. aegypti*, the anterior region of medium midgut was recovered by a continued PE. However, morphological observation of larvae submitted to aqueous extract of *I. suffruticosa* leaves showed disruption on the PE structure. Clearly, we are far away from completely elucidating the mechanisms of *I. suffruticosa* to induce growth retardation in animal models. However, studies from our group also demonstrated that this plant is an extremely powerful inducer of cancer cell death and possibly the bioactive compound from *I. suffruticosa* could act binding many molecular targets inside the cell activating alternative apoptotic pathways or inducing mitotic catastrophe which indicates a form of cell death that is caused by aberrant mitosis by caspase 3 activation and oligonucleosomal DNA degradation [24]. On the whole, all the aforementioned data indicate that *I. suffruticosa* can induce cell death via different molecular pathways and with different executing mechanisms, that is classical apoptosis, but also mitotic catastrophe. These activities and the main recognized molecular targets of *I. suffruticosa* are depicted in Figure 3. Due to these actions, *I. suffruticosa* can impinge upon different conditions (represented as circles in the Figure 3).

Plants and their derivatives were used for controlling and eradicating mosquitoes and other domestic pests before the advent of synthetic organic chemical [21].

The use of plant extracts in insects control is an alternative pest control method for minimizing the noxious effects of some pesticide compounds on wildlife, livestock, non target insect species, and the environment [25].

There is a general lack of effective and inexpensive chemotherapeutic agents for treating this disease that occurs in the developing world. In addition, specimens from sites where there has already been intensive use of the larvicide in dengue control programs are more likely to show resistance to the larvicide, and it has become a severe problem [26]. 

In this sense, new insecticides of herbal origin discovered through ethnopharmacological studies have shown interesting results. Our laboratory has initiated and developed original investigations, and we have evaluated the embryotoxicity caused by compounds from natural extracts of plants.

Purification of the bioactive component(s) from *Indigofera suffruticosa* is underway, and further investigations may improve our understanding of possible developmental changes from aqueous extract of this plant used in folk medicine.

## Figures and Tables

**Table 1:** Effect of aqueous extract of *I*. *suffruticosa* leaves in different concentrations on development of eggs of *Aedes aegypti*.

Eggs^a^	Days	Treated^b^				Control^c^
Eclosion (%)		Concentration (*μ*g/mL)				
		250	500	750	1000	
	0–7	0.0^d^	0.0^d^	0.0^d^	0.0^d^	35

^a^No. of eggs = 40; ^b^aqueous extract of leaves of *I. Suffruticosa;* ^c^distilled water; ^d^No eclosion.

**Table 2:** Inhibitory effect of aqueous extract of *I*. *suffruticosa* leaves in different concentrations on development of the first instar larvae (L1) of *Aedes aegypti*.

Larvae L1^a^	Days	Treated^b^				Control^c^
Inhibition (%)		Concentration (*μ*g/mL)				
		250	500	750	1000	
	0	0.0	0.0	0.0	0.0	0.0
	24	6.6	13.3	40.0	20.0	40.0
	48	100.0	40.0	100.0	60.0	73.0
	72	93.3	20.0	53.3	46.6	46.0

^a^No. of larvae = 15; ^b^aqueous extract of *I*. *suffruticosa* leaves; ^c^distilled water.
